# Response to the “Letter to the Editor” by Gabor Mezei et al., Comments on Vimercati et al., 2019, “Asbestos exposure and malignant mesothelioma of the tunica vaginalis testis: a systematic review and the experience of the Apulia (Southern Italy) mesothelioma register”

**DOI:** 10.1186/s12940-019-0553-8

**Published:** 2019-12-26

**Authors:** Luigi Vimercati, Domenica Cavone, Maria Celeste Delfino, Luigi De Maria, Antonio Caputi, Giovanni Maria Ferri, Gabriella Serio

**Affiliations:** 1 Interdisciplinary Department of Medicine (DIM), Unit of Occupational Medicine, University Aldo Moro of Bari Medical School, 11 G. Cesare Square, 70124 Bari, Italy; 2 Department of Emergency and Organ Transplantation (DETO), Pathology Division, University Aldo Moro of Bari Medical School, 11 G. Cesare Square, 70124 Bari, Italy

**Keywords:** Malignant mesothelioma, Tunica vaginalis testis, Epidemiology, Etiology

Dear Sir,

Thank you for the opportunity to respond to the Letter to the Editor you have received from Gabor Mezei et al., on our study titled “Asbestos exposure and malignant mesothelioma of the tunica vaginalis testis: a systematic review and the experience of the Apulia (southern Italy) mesothelioma register” [1].

We thank the Authors for their comments, which open the debate that we aimed at by submitting our paper. Here, we provide our specific comments regarding their concerns.

They highlight that our review incorrectly states that *“the most recent IARC Monograph on the carcinogenic risks of asbestos (IARC, 2012) in support of their view that asbestos is an established cause of MMTVT.”* The criticism is correct, and we thank them for pointing out that our suggestion in reference [1] is not correct.

However, it is important to note that confounding due to exposure to asbestos is always a concern when studying mesothelioma. Regarding the rare or non-existing cases of MMTVT reported in occupational cohorts and the absence of temporal or geographical trends corresponding with commercial asbestos use, we consider it necessary to keep in mind the rarity of this disease and at the same time the relatively recent agreement on the definition of the clinicopathological entity [1], which may have involved difficulty in diagnosis, miss-classification and miss-coding. Moreover, registry-based studies on cancer might be affected by detection bias (also called surveillance bias) [2].

Lowry and Weiss, cited in the letter, mention that *“this study was limited by small numbers and was unable to directly ascertain asbestos exposure”* [3].

We disagree with the statement that only heavy workplace exposures to asbestos may induce MMTVT; proof of this is, for example, the occurrence of peritoneal mesothelioma in subjects with only established environmental exposure [4].

Unfortunately, earlier studies on 289 MMTVT cases reported that 47.6% of the cases with asbestos exposure investigated are positive, whereas for 42% of cases, these data are not available, i.e., have not been investigated (see table 2 in Vimercati et al. 2019 [1]).

Regarding the cited study of Marinaccio et al. 2010 [5], of which some of us are co-authors, we would like to note that in table 3 of the work, 50% of MMTVT cases are reported in economic sectors such as in the construction, steel, and transport construction and maintenance industries wherein the use of asbestos is well known in the technological cycles as well as (especially in past years, and this agrees with the latency time) the failure to comply with safety requirements to protect the health of workers [6].

More recently, as we reported in our review, in the sixth report of the Italian National Mesothelioma Register (Renam) on 68 cases of MMTVT recorded in 1993–2015, 78% of cases were ascertained as exposure to asbestos [7].

We also agree with the Authors that case reports and case series are not epidemiological studies, but, as mentioned above, we do not agree with the Authors statement *“cases reported in the published literature did not have documented asbestos exposure”, as* exposure to asbestos is not “undocumented” but is not investigated at all, as we have seen in our review paper [1].

We disagree with the statement that *“there is a considerable evidence that in addition to asbestos…. ionizing radiation increase the risk of malignant mesothelioma”,* which in our opinion cannot be applied to MMTVT despite the biological plausibility in the current state of knowledge. To the best of our knowledge, only more cases of pericardial mesothelioma after therapeutic radiation have been reported [8–11], but not MMTVT. Of note, the same Authors, Mezei et al. (2017), reported that “*for mTVT , prior history of radiation therapy was nonexistent”* [12].

Finally, in regards to “*the current paradigm of carcinogenesis”,* we agree with Authors and with the current knowledge on a background incidence of non-asbestos-related mesothelioma or idiopathic cases, as well as with the possible role of talc, environmental exposures, genetic predisposition and gene-environment interactions in the development of this disease [13–18].

Similarly, we agree with the Authors that little is known about the epidemiology, the amount of information on potential confounders is limited, and the aetiology of MMTVT is inadequately researched and poorly understood.

However, there is growing evidence on the potential aetiologic role of asbestos exposure in MMTVT development, although the underlying mechanisms remain to be elucidated. While not definitive evidence of causality, considering both case reports and mode of action, it is certainly suggestive.

It should be emphasized that our cases, as mentioned in the paper, have established an ascertained exposure to asbestos, an average latency period of 47 years, have no other cancers and have ever been subjected to radiation therapy or exposed to ionizing radiation.

As stated in our discussion, the preliminary results of the case control study from the Italian mesothelioma registry, by Marinaccio et al. personal communication, show that occupational exposure to asbestos was significantly associated with the risk of the disease (OR = 7.940; 95% CI 3.01–18.2 in MMTVT).

In summary, the weight of evidence from currently available data strongly suggests that asbestos exposure increases mesothelioma TVT risk.

Further contributions from a more in-depth assessment are needed to promote a substantial improvement in the knowledge of the epidemiology of MMTVT.

## Data Availability

All references are publicly available.

## Abbreviations

IARC: International agency research on cancer
MMTV: Malignant mesothelioma tunica vaginalis testis
OR: Odds ratio
TVT: Tunica vaginalis testis
